# Assessing Changes in the Distribution Patterns of the European Wildcat in Hungary

**DOI:** 10.3390/ani14050785

**Published:** 2024-03-02

**Authors:** Chimed Otgontamir, Ádám Fehér, Gergely Schally, Davaa Lkhagvasuren, Zsolt Biró

**Affiliations:** 1 Department of Wildlife Biology and Management, Institute for Wildlife Management and Nature Conservation, Hungarian University of Agriculture and Life Sciences, Páter Károly u. 1, H-2100 Godollo, Hungary; tamir.urin@yahoo.com (C.O.); schallygergo@gmail.com (G.S.); 2 Department of Biology, School of Arts and Sciences, National University of Mongolia, WWF9+6H6, Ulaanbaatar 14200, Mongolia

**Keywords:** *Felis silvestris*, wildcat occurrence, wildcat distribution, wildcat conservation, Hungary

## Abstract

**Simple Summary:**

We investigated the nationwide distribution of the European wildcat (*Felis silvestris* Schreber, 1777) in Hungary during three time periods (2004, 2014, and 2022) to assess changes in its distribution and to examine the impact of land cover variables on its occurrence across the country. An online questionnaire survey of Hungarian game management units revealed that the distribution of the species has increased over the last two decades in Hungary, with the eastern, northern, and south-western parts of the country holding the majority of its population. In addition, the results indicated that the presence of the species was significantly higher in areas with high broad-leaved forest cover. From this study, we conclude that the wildcat population showed a positive trend; however, the population vanished in some parts of Hungary, and broad-leaved forests are the most important habitat for wildcats.

**Abstract:**

The European wildcat (*Felis silvestris* Schreber, 1777) is an endangered and elusive carnivore that is slowly recovering in Central Europe after persecution and a decline in its distribution over the past two centuries, and specific conservation plans are needed in most of its range. Knowledge of the continent-wide distribution and status of this species is still poor. Using an online questionnaire, we evaluated the nationwide distribution of wildcats across three time periods (2004, 2014, and 2022) in Hungary. The species’ reported occurrence was analyzed according to binominal logistic regression using the percent cover of land cover categories as explanatory variables. We found that the spatial cover of broad-leaved forest was positively associated with the occurrence of wildcats, and the analysis revealed a positive trend in the larger 2004–2022 time frame. We also recorded that although wildcats have disappeared from areas of the central, southern, and western parts of Hungary, regions in the eastern, northern, and south-western areas appear to retain stable populations.

## 1. Introduction

The European wildcat (*Felis silvestris*) is a small-sized native felid living across Europe [1,2], with its most contiguous distribution in Germany and its adjacent areas [3,4]. Traditionally considered to be a forest specialist [2], recent studies support its preference for a wide range of habitats, such as a variety of forests that connect open areas, grasslands, meadows, scrublands, agriculture, and pastures [3,5,6,7,8].

The wildcat is currently classified as Least Concern on the IUCN Red List, and the primary threats to wildcat populations include habitat fragmentation, hybridization with feral cats (*Felis silvestris catus*), illegal hunting, trapping, traffic mortality, diseases carried by domestic cats, and climate change [2,4,9,10,11]. In Europe, the species also faces the challenge of our limited ecological knowledge of it due to its inconspicuous behavior. This includes scarce information on abundance, mortality, and regional distribution across most of its range. Moreover, detailed insights into the occurrence and potential presence of the species are notably lacking, remaining understudied and contentious [12,13].

Understanding species distributions and identifying threats and determining their occurrence are pivotal aspects of conservation efforts, particularly for species that are endangered and difficult to detect [14,15]. The wildcat is one of Europe’s most endangered carnivores; moreover, in Hungary, its distribution has shrunk, most of its former range has vanished, and it has permanently disappeared from some areas over the last decade [2,5,11]. In Hungary, previous surveys performed by Heltai et al. [5] revealed that wildcat distribution had declined and is completely restricted to the country’s northwest and central areas. The results show that the Great Plain, Transdanubia, the Mecsek Mountains, and the North Hungarian Mountains are home to a stable population of wildcats [5]. The wildcat has been strictly protected since 2012, and long-term conservation and management for the species were recommended by Stahl and Artois [13]. One crucial proposal recommended regular monitoring of the distribution of the wildcats.

In this study, we used online questionnaire data collected from game management units (GMUs) to (1) assess the present wildcat distribution in Hungary at a broad scale and analyze alterations in its distribution between 2004 and 2022 and (2) examine the impact of characteristic land cover types on wildcat occurrence across the country. A robust understanding of how the species distribution changes over time and the variables that influence these changes provide essential data for identifying priority areas for long-term persistence and effective conservation of this elusive species.

## 2. Materials and Methods

### 2.1. Survey Design and Data Collection

In Hungary, various research projects have been performed to study the occurrence of wildcats through game management units (GMUs) in the years 2004, 2014, and 2022. Questionnaires were distributed to the managers of GMUs via email, and the respondents replied within 1–1.5 months. The survey included questions on sightings of wildcats in the given management areas in three time periods (2004, 2014, and 2022). We considered instances of direct sightings and clearly identifiable pugmarks and scrapes to be species detections. The reported wildcat occurrence was evaluated nationwide at a broad scale using the Universal Transverse Mercator (UTM) coordinate system covering the study area over Hungary with grid cells of 10 × 10 km each, representing an area similar to the estimated maximum distribution area of wildcats according to European mammal mapping [1]. All Hungarian GMUs have an individual code provided by the Hungarian Game Management Database that is connected to every respondent [16], and we localized the data geographically using the UTM cells.

We used 6.25 km^2^ of coverage as the detection threshold in each grid cell, which was selected by Heltai et al. [5] based on the information provided by the Hungarian Hunting Act, which states that the allowed size of a game management unit is 30 km^2^. However, in the worst scenario, the smallest hunting area might be 25 km^2^, and this area can be divided into four equal 6.25 km^2^ parts. We aimed to continue the previous methodology for ease of comparison and considered that if we had information from at least 6.25% of a UTM grid cell on wildcat presence, then we could confirm the detection of wildcats in the total area of the relevant UTM cell. Consequently, if the area of one or more game management units reporting wildcat presence reaches a total coverage of 6.25 km^2^ inside one UTM cell, a valid detection can refer to the total area of the cell. Multiple detections of wildcats in the same grid cell were merged into one detection for that grid cell and time period.

### 2.2. Data Analysis

We created detection histories for each grid cell and each sampling period by creating a dichotomous variable on wildcat occurrence, where a value of 1 indicated the presence and of 0 the absence of the species in the UTM cell based on the responses. We hypothesized that the wildcats would typically use forested areas and open areas with water bodies, which are strongly linked to habitat use and believed to provide shelter [3,6,17,18]. Furthermore, we also hypothesized that wildcats avoid human settlements due to noise, light, dogs, and humans presence [6]. To test this hypothesis, we used the CORINE Land Cover database (*CORINE Land Cover*, 2006, 2012, 2018) [19,20,21] for each corresponding survey year to find and evaluate which habitat types influenced wildcat occurrence. Seven different land cover types were selected, which are the most common and dominant land cover classes throughout Hungary (Table 1).

The percent cover of land cover classes was calculated relative to the total area of each relevant UTM cell and used as an explanatory variable in the statistical analysis. Additionally, we calculated the richness of the land cover types by counting the available CLC classes per UTM cell and the evenness, Shannon–Wiener, and inverse Simpson diversity indices of the land cover types based on their areal data. Wildcat occurrence was analyzed as a function of these explanatory variables using binomial logistic regression in R [22]. The best model structure was chosen using likelihood ratio tests to select which variable was a significant predictor of wildcat occurrence. In addition, we directly compared the area of the land cover classes between UTM cells with and without wildcat occurrence by performing Welch’s two-sample t-tests. The figures were created using the *ggplot2* [23] and *ggpubr* [24] packages, while the diversity indices were calculated using the *vegan* package [25] in R. We created maps to visualize the presence and absence of wildcats in the three time periods using QGIS v. 3.32.3.

## 3. Results

The game management units reported the absence and presence of wildcats in three distinct time periods, with a notable decline in responsiveness observed from 2004 to 2022. The rate of positive answers was correlated with the total number of responses, but due to low number of repetitions, it remained non-significant (r = 0.93, *p* = 0.28). Although the number of respondents decreased over time, this sample loss was spatially balanced. Therefore, the number of covering UTM cells of interest remained stable during the study (Table 2), which provided the sampling units to our analyses instead of direct responses. The area represented by the relevant UTM cells was 1.801 thousand hectares in 2004 and exceeded 1.985 thousand hectares in 2014 and 2022.

**Figure 1:**
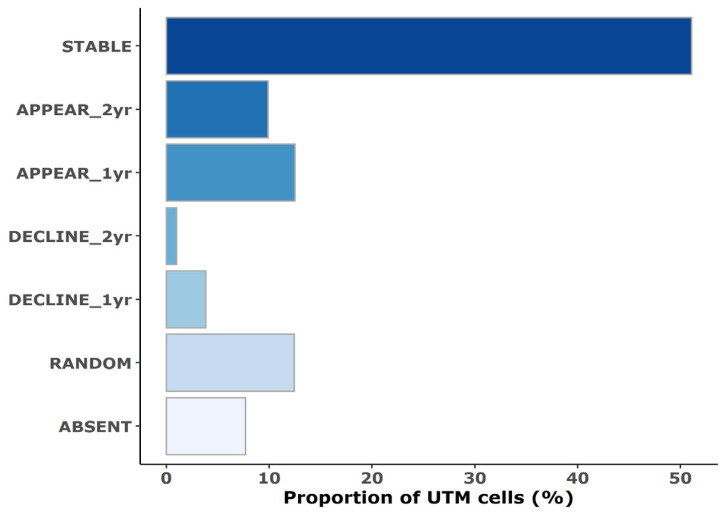
Trends in wildcat occurrence during the survey’s time frame based on the relative proportion of the studied UTM cells. **STABLE** = wildcats were reported present in each survey year, **APPEAR_2yr** = wildcats were reported present in the last two survey years, **APPEAR_1yr** = wildcats were reported present in the last survey year only, **DECLINE_2yr** = wildcats were reported present in the first two survey years only, **DECLINE_1yr** = wildcats were reported present in the first year of the survey only; **RANDOM** = wildcats temporarily disappeared or reappeared in 2014, **ABSENT** = wildcats were reported absent in each survey year.

The increase in wildcat detections was also reflected in the UTM grid scale. The majority of the studied cells had at least one GMU connected to it that reported wildcat detection in each survey year (Table 2). The covered area of detections slightly increased from 1.207 thousand ha to 1.304 thousand ha between 2004 and 2014 and reached 1.688 thousand hectares in 2022. Overall, almost half of the studied UTM cells (51.1%) could be coded as “occupied” by wildcats based on the relevant detections of the corresponding GMUs (Figure 1), indicating a stable presence. Wildcats appeared present starting from 2014 in 9.9% of the cells and were only detected for the first time in 2022 in 12.5% of the UTM cells.

**Figure 2:**
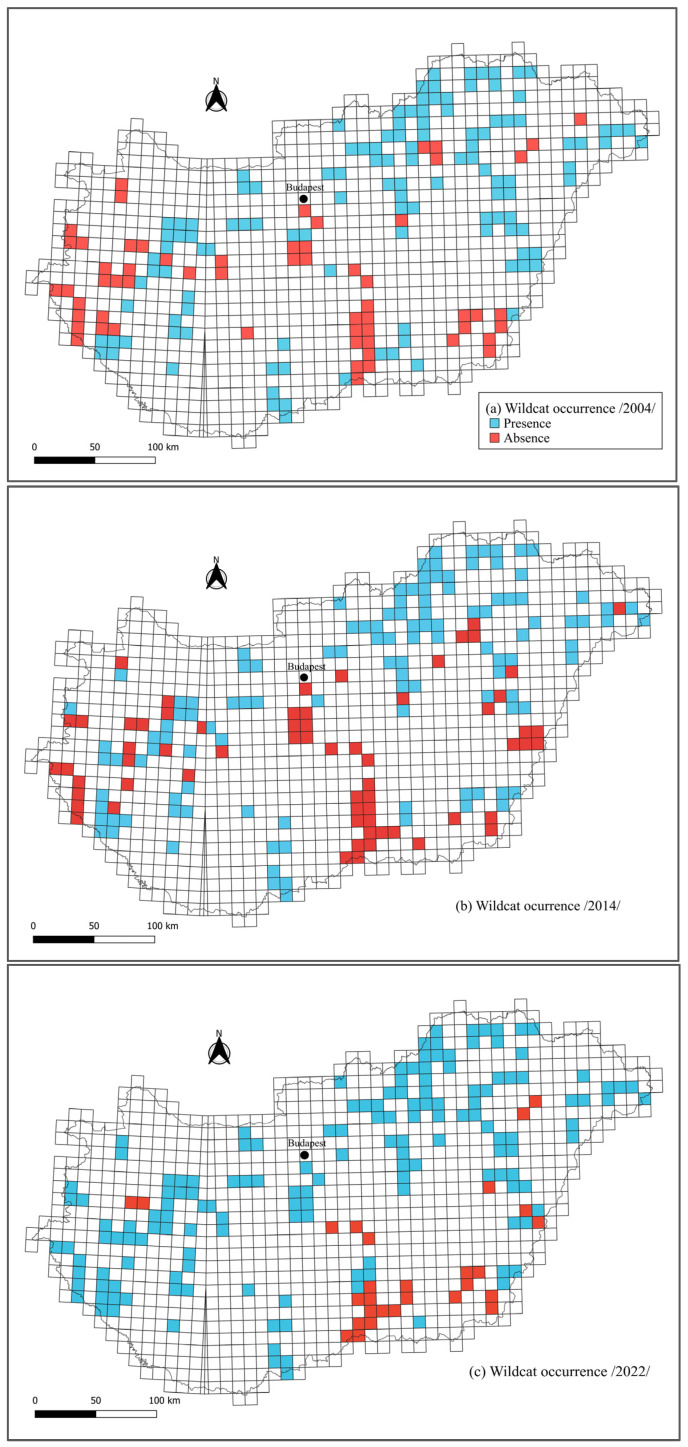
Reported occurrence of wildcats in Hungary between 2004 and 2022 (**a**–**c**).

The number of regions in which wildcats disappeared was slightly lower: after nearly two decades, wildcats were reported absent in 2022 in 1% of the grid cells, while 3.8% of the cells had wildcat detections only from 2004. There were many GMUs and thereby UTM cells (12.4%) in which the wildcat detections were quite sporadic: wildcats were detected in 2014 in contrast with their absence in the previous and subsequent survey years or reappeared after a temporary absence in 2014. Consecutive absence was reported in 7.7% of the UTM cells (Figure 2).

The most common and dominant land use type was non-irrigated arable land throughout the studied UTM cells, with a mean area of 4.736 ± 2.404 ha and 48 ± 24% cover. The average area of broad-leaved forest was also high (1.942 ± 1.845 ha) but less dominant in the UTM cells (19 ± 19%), while pasture was the third most frequent land cover type, with a much smaller mean area (749 ± 509 ha) and cover (7 ± 5%). The mean cover of the other land cover types remained under 5%. The studied UTM cells had four or five different land cover types present inside their area on average (mean: 4.64 ± 1.5). The diversity of the available land cover types remained nearly the same across all time periods (Shannon–Wiener index: 0.83 ± 0.36; inverse Simpson index: 2.04 ± 0.78; evenness: 0.54 ± 0.19), indicating relatively low variability over the years. During the model selection, likelihood ratio tests revealed that the diversity indices were not good predictors of wildcat occurrence (richness of land cover types: χ^2^ = 0.01, *p* = 0.93; evenness: χ^2^ = 0.29, *p* = 0.58; Shannon diversity: χ^2^ = 0.16, *p* = 0.69). The survey years and the percent cover of the land cover types constituted the final set of explanatory variables.

The coefficients of the fitted logistic regression model were expressed in their exponentiated form, resulting in a more comprehensible odds ratio (OR). This metric ranges from 0 to infinity, where OR = 1 functions as a threshold to divide negative and positive associations. If 0 < OR < 1, the preferred event of interest (i.e., wildcats are present) is less likely to occur; if 1 < OR, the preferred event is more likely to occur. Values farther from 1 in a given direction represent a stronger association [26]. The logistic regression model found a significantly increasing trend in the larger time frame between 2004 and 2022 in wildcat detections (Table 3). Among the land cover types, broad-leaved forest cover was positively associated with wildcat occurrence, but the estimated odds remained low nevertheless, staying near the threshold of 1 (OR = 1.06, 95% confidence interval: 1.03–1.08). On the contrary, mixed forest cover (woodlands where the standing volumes of coniferous and broad-leaved tree species were nearly equal) turned out to be negatively associated with wildcat presence but also with a weak OR. Non-irrigated arable land as the most dominant land cover type in the vast majority of the UTM cells had no effect on wildcat occurrence, similar to the remaining minority land cover types (Table 3).

**Figure 3:**
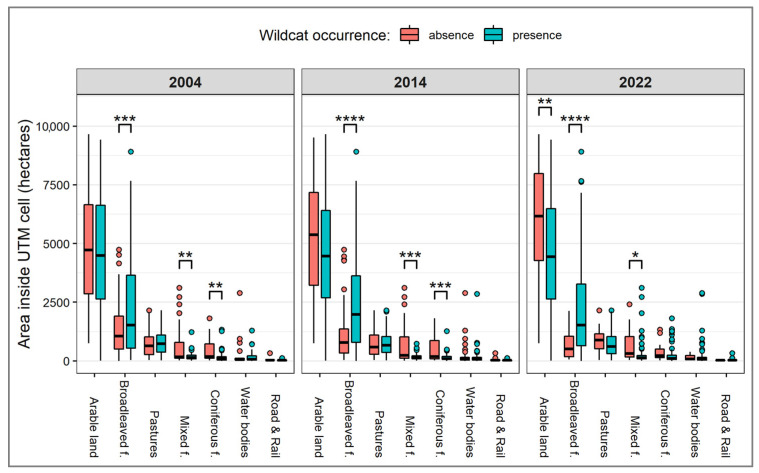
Box plot comparing the area of land cover types in UTM cells with and without reported wildcat presence. Asterisks represent the significant pairwise differences indicated using Welch’s two-sample t-test. **** *p* < 0.0001, *** *p* < 0.001, ** *p* < 0.01, * *p* < 0.05.

For each survey year, the area of broad-leaved forest was significantly higher (*p* < 0.001) in the UTM cells with wildcat presence (2004: 2.215 ± 2.073 ha; 2014: 2.384 ± 1.967 ha; 2022: 2.149 ± 1.888 ha) than those without it (2004: 1.370 ± 1.192 ha; 2014: 1.062 ± 1.111 ha; 2022: 671 ± 528 ha). The percent cover of this habitat reflected the same contrast between occupied (2004: 22 ± 21%; 2014: 23 ± 20%; 2022: 21 ± 19%) and non-occupied (2004: 13 ± 12%; 2014: 10 ± 11%; 2022: 6 ± 5%) grid cells. Mixed forests had a higher area in non-occupied cells (2004: 602 ± 786 ha; 2014: 659 ± 808 ha; 2022: 630 ± 668 ha) than in those where wildcats were present (2004: 189 ± 168 ha; 2014: 169 ± 125 ha; 2022: 263 ± 454 ha), supporting the findings of the logistical regression model. The same tendency was true in the case of coniferous forests in 2004 and 2014 (Figure 3), but their effect on wildcat occurrence was not confirmed using the model.

## 4. Discussion

The population of wildcats in Europe has recovered slowly, particularly in Central Europe and Italy [6,12], whereas in the Iberian Peninsula and in Scotland, the population is declining [27,28].

Our study suggests that the distribution area of wildcats in Hungary has increased over the past two decades, and the bulk of their distribution is across the Great Plain, the North Hungarian Mountains, and the Transdanubian Mountains. These results are similar to earlier studies showing stable wildcat populations in the forests of the floodplains in the Great Plain, as well as in the Dráva Plain and the Mecsek, Villányi, Transdanubian, and North Hungarian Mountains [5]. In contrast, we found no occurrence of wildcats in the central and western parts of Hungary or some parts of southern Hungary in the three distinct time periods either, in accordance with the results of Heltai et al. [5], who recorded that the species had disappeared from many areas of Hungary, particularly from the central and north-western regions.

Typically, wildcats are considered a forest species [2]. The occurrence of the wildcat in Hungary, at a broad scale, was best explained by broad-leaved forest cover. In other words, broad-leaved forest cover considerably increased the probability of wildcat detection. This result is supported by previous studies in which, for example, Mattucci et al. [29] mentioned that the distribution of European wildcats is supported by areas of broad-leaved forests around the Mediterranean. On the other hand, this species is regarded a habitat generalist [8]: wildcats appear to use a wide range of habitats. Studies, especially in western Europe (e.g., Germany and Scotland), have shown that their presence is linked to coniferous forests, grasslands, and scrubland and is limited by forest, forest ecotone, and meadow [6,30,31]. Whereas, in Mediterranean countries, scrub areas are thought to be essential habitats for their distribution [32]. Nevertheless, we found that broad-leaved forest is the most important habitat for wildcats, which has a positive impact on their distribution at a broad scale. This finding is supported by an earlier small-scale investigation carried out in Hungary [3].

Mixed and coniferous forests showed a negative association with their probable occurrence, as previously recorded by Silva et al. [8]. They supposed that the occurrence of wildcats in Scotland could be affected by categorizing the woodland into different groups, such as mixed and coniferous forests. Likewise, we also assessed these land cover types as independent variables to examine the occurrence of wildcats in Hungary, which may have influenced their negative association with the species.

Wildcats avoid pasture areas because grassland-covered areas are frequently connected to agricultural areas, where farmers are often present and which are less suitable for small mammals and rodents due to being intensively grazed [33]. However, richly structured agricultural landscapes can be inhabited by wildcats, including for their successful reproduction [34]. In contrast, wildcats use open pastures and cattle pastures, which have an important role in wildcats hunting, as well as these areas contributing to a higher prey density, like that of the montane water vole [35,36]. We detected no significant association of pasture areas with wildcat occurrence in Hungary.

We did not detect any influence of water bodies on wildcat occurrence. Water courses have been considered a crucial factor for wildcat occurrence and may have a significant impact regionally [6]. Typically, water courses are linked with riparian habitats, which often have a wide variety of prey [37]. Nonetheless, we found a negative association between wildcat occurrence and water bodies at a broad scale.

Wildcats avoid residential areas such as roads [6], rail networks, and associated land, which maycontribute to probable wildcat absence. Similarly, Silva et al. [8] found no evidence that urban areas and roads were influential in wildcat presence. Even they avoided human settlements based on the radiotelemetry data in Hungary [3]. This might be due to the presence of dogs, feral cats, and humans, as well as a combination of light and noise, which influence their spatial behavior [6].

## 5. Conclusions

We conclude from our results that the European wildcat is associated with broad-leaved forest habitats at the national level, and there seems to be a general increase in its distribution over the past two decades. However, we recognize the need for further study to fully understand its habitat and distribution at a broader scale. Therefore, to retain and safeguard its habitat and expand the population of wildcats in Hungary, conservation efforts should be focused toward habitat management interventions, including maintaining and conserving broad-leaved forest cover areas and core habitats where a stable population is presented. These results do, however, highlight the importance of monitoring this elusive cat to inform local and regional conservation strategies and action plans.

## Figures and Tables

**Table 1:** List of the used CORINE Land Cover (CLC) classes.

CLC Main Category	CLC Class Code	Description
Artificial surfaces	122	Road and rail networks and associated land
Agricultural areas	211	Non-irrigated arable land
	231	Pastures
Forest and semi-natural areas	311	Broad-leaved forest
	312	Coniferous forest
	313	Mixed forest
Water bodies	512	Water bodies

**Table 2:** The total number of respondents and the related UTM cells per survey year with the relative proportion of reported wildcat occurrence.

	2004		2014		2022	
Respondents	n	presence %	n	presence %	n	presence %
	551	52	354	51	200	86
UTM cells	182	67	201	66	201	85

**Table 3:** Temporal changes in reported wildcat presence and effects of land cover types on wildcat occurrence estimated using binomial logistic regression. The year 2004 was used as reference category for the variable Year. The coefficients and their corresponding confidence intervals are expressed in odds ratios, the exponentiated form of the default log of the odds output of the model.

	95% Confidence Interval				
	Estimate	Lower	Upper	Z Value	*p*
**Intercept**	1.50	0.44	5.12	0.65	0.519
**Year**					
2014	0.92	0.57	1.47	−0.35	0.724
2022	3.47 ***	1.99	6.03	4.4	<0.001
**Relative proportion of land cover types**					
Roads, rail, and associated land	0.60	0.29	1.25	−1.36	0.172
Non-irrigated arable land	0.99	0.98	1.01	−0.78	0.433
Pastures	1.02	0.98	1.07	1.05	0.295
Broad-leaved forest	1.06 ***	1.03	1.08	4.77	<0.001
Coniferous forest	0.92	0.80	1.06	−1.13	0.260
Mixed forest	0.87 *	0.78	0.97	−2.43	0.015
Water bodies	1.00	0.94	1.06	0.00	0.999

*** *p* < 0.001 * *p* < 0.05.

## Data Availability

The data presented in this study are available upon request from the authors.
