# Correction: Otgontamir et al. Assessing Changes in the Distribution Patterns of the European Wildcat in Hungary. *Animals* 2024, *14*, 785

**DOI:** 10.3390/ani14132008

**Published:** 2024-07-08

**Authors:** Chimed Otgontamir, Ádám Fehér, Gergely Schally, Miklós Heltai, László Szabó, Róbert Lehoczki, Davaa Lkhagvasuren, Zsolt Biró

**Affiliations:** 1 Department of Wildlife Biology and Management, Institute for Wildlife Management and Nature Conservation, Hungarian University of Agriculture and Life Sciences, Páter Károly u. 1, H-2100 Godollo, Hungary; tamir.urin@yahoo.com (C.O.); heltai.miklos.gabor@uni-mate.hu (M.H.); szabo.laszlo@uni-mate.hu (L.S.); 2 Department of Biology, School of Arts and Sciences, National University of Mongolia, Ulaanbaatar 14200, Mongolia; 3 Lechner Knowledge Center, Budafoki út 59, H-1111 Budapest, Hungary; robert.lehoczki@lechnerkozpont.hu

## Update Affiliations

In the original publication [1], there was an error regarding the affiliation for Chimed Otgontamir and Davaa Lkhagvasuren. In affiliation 2, there was unnecessary text; the updated affiliation should be: Department of Biology, School of Arts and Sciences, National University of Mongolia, Ulaanbaatar 14200, Mongolia. The third affiliation “Lechner Knowledge Center, Budafoki út 59, H-1111 Budapest, Hungary” is added.

## Addition of Authors

Miklós Heltai (M.H.), László Szabó (L.S.), and Róbert Lehoczki (R.L.) were not included as authors in the original publication. The corrected Author Contributions statement appears here.

**Author Contributions:** Conceptualization, Z.B. and C.O.; methodology, Z.B., Á.F., G.S., D.L. and C.O.; software, Á.F., G.S. and C.O.; investigation, Z.B., C.O., M.H. and L.S.; data curation, C.O., Á.F., Z.B., L.S. and R.L.; writing—original draft preparation, Á.F., Z.B. and C.O.; writing—review and editing, C.O., Z.B., Á.F., G.S., D.L., M.H., L.S. and R.L.; visualization, C.O., Á.F., Z.B. and R.L.; supervision, Z.B. and D.L. All authors have read and agreed to the published version of the manuscript.

## Text Correction

There was an error in the original publication. The first questionnaire survey in 2004 was offline and sent to the recipients via postal mail.

A correction has been made to the Introduction section, paragraph 4:

In this study, we used offline (paper) and online questionnaire data collected from game management units (GMUs) to (1) assess the present wildcat distribution in Hungary on a broad scale and analyze alterations in its distribution between 2004 and 2022 and (2) examine the impact of characteristic land cover types on wildcat occurrence across the country. A robust understanding of how the species’ distribution changes over time and the variables that influence these changes provides essential data for identifying priority areas for the long-term persistence and effective conservation of this elusive species.

A correction has been made to the Materials and Methods section in the Survey Design and Data Collection subsection, paragraph 1:

In Hungary, various research projects have been performed to study the occurrence of wildcats through game management units (GMUs) in the years 2004, 2014, and 2022. Questionnaires were distributed to the managers of GMUs via postal mail and email, and the respondents replied within 1–1.5 months. The survey included questions on sightings of wildcats in the given management areas in three time periods (2004, 2014, and 2022). We considered instances of direct sightings and clearly identifiable pugmarks and scrapes to be species detections. The reported wildcat occurrence was evaluated nationwide on a broad scale using the Universal Transverse Mercator (UTM) coordinate system, covering the study area over Hungary with grid cells of 10 × 10 km each, representing an area similar to the estimated maximum distribution area of wildcats according to European mammal mapping [1]. All Hungarian GMUs have an individual code provided by the Hungarian Game Management Database that is connected to every respondent [16], and we localized the data geographically using the UTM cells.

The authors state that the scientific conclusions are unaffected. This correction was approved by the Academic Editor. The original publication has also been updated.
